# Non-invasive molecular tracking method that measures ocular drug distribution in non-human primates

**DOI:** 10.1038/s42003-019-0731-9

**Published:** 2020-01-08

**Authors:** Guillaume Normand, Michael Maker, Jan Penraat, Kellyann Kovach, Joy G. Ghosh, Cynthia Grosskreutz, Sudeep Chandra

**Affiliations:** 1 Department of Translational Medicine, Novartis Institutes for BioMedical Research, East Hanover, NJ 07936 USA; 2 Lab Animal Services, Novartis Institutes for BioMedical Research, East Hanover, NJ 07936 USA; 3 Ophthalmology Research, Novartis Institutes for BioMedical Research, Cambridge, MA 02139 USA; 4 Present Address: InviCRO, 27 Drydock Avenue, Boston, MA 02210 USA; 5 Present Address: PetSmart, 33963 Doheny Park Road - San Juan, Capistrano, CA 92675 USA; 6 Present Address: Pfizer Corporation, Comparative Medicine, Middletown Road, Pearl River, NY USA; 7 Present Address: Bain Capital Life Sciences, 200 Clarendon Street, Boston, MA 02116 USA; 8 Present Address: Ophthalmology Research, Novartis Institutes for Biomedical Research, Cambrodge, MA 02139 USA

**Keywords:** Drug delivery, Optical imaging

## Abstract

Intravitreal (IVT) injection has become the standard route for drug administration in retinal diseases. However, the ability to measure biodistribution of ocular therapeutics in large species remains limited, due to the invasive nature of some techniques or their lack of spatial information. The aim of this study was to develop in cynomolgus monkeys a non-invasive fluorescence imaging technology that enables tracking of IVT-dosed drugs and could be easily translated into humans. Here, we show a proof-of-concept for labeled ranibizumab with observed half-lives of 3.34 and 4.52 days at the retina and in the vitreous, respectively. We further investigate a long acting anti-VEGF antibody, which remains as an agglomerate with some material leaking out until the end of the study at Day 35. Overall, we were able to visualize and measure differences in the in vivo behavior between short and long-acting antibodies, demonstrating the power of the technology for ocular pharmacokinetics.

## Abstract (web summary)

Guillaume Normand et al. present a non-invasive fluorescence imaging technology that enables a longitudinal tracking of drugs delivered into the eyes. This method allows direct monitoring of any drugs delivered into the eyes, which could potentially inform clinicians of optimal dosing frequency for each patient.

## Introduction

The success of anti-vascular endothelial growth factor (anti-VEGF) therapy and its use as first-line treatment for neovascular age-related macular degeneration has made intravitreal (IVT) injection a common dosing technique to deliver biologics in the eye^1,2^. In contrast to intravenous dosing, this approach allows direct drug delivery at an optimal dose to the target tissue and limits potential systemic exposure. However, IVT dosing poses some unique challenges for drug development in the clinic, since standard pharmacokinetic methods cannot be implemented for the posterior segment of the eye. The general approach in drug development for such measurements has thus been to conduct animal studies postmortem to experimentally determine the vitreal and retinal distribution of biologics^3–5^ and predict the distribution in humans through modeling. However, the bioanalytical methods employed cannot be implemented in humans due to the invasive nature of tissue access in the eye. Furthermore, these assays often lack spatial information (due to homogenized tissue-based measurements) and provide low temporal resolution (only a few time points are usually analyzed). With the advent of novel approaches to extend the retention time of biologics at the retina, such as PEGylated or hyaluronan-binding antibodies^6,7^, it is imperative to develop novel non-invasive translatable platforms that can directly determine the ocular distribution of long-acting agents in the human eye.

The goal of this study was to develop a non-invasive imaging platform that would allow longitudinal tracking of any IVT-injected therapeutics in the living eye. Previously, similar work has been attempted using positron emitted tomography^8^ and magnetic resonance imaging^9,10^. Although these imaging techniques offer value, they are translationally not very useful for many reasons. First, these imaging modalities need either complex instrumentation or radioactivity, which pose unique challenges and safety concerns for clinical practice in ophthalmology, where radiological tools are rarely accessible. Second, the spatial resolution of magnetic resonance imaging and positron emitted tomography in the eye is usually lower, which makes it difficult to measure concentrations in discrete compartments, for instance, to differentiate the retina from the vitreous. Third, these technologies are expensive and require non-ophthalmic expertise to acquire appropriate scans, reducing the chance of widespread clinical adoption. Finally, as long-acting agents are designed to reside over few months in man, drug tracking would require a stable non-radioactive reporter with a long half-life. Thus the most relevant choice was to develop an optical tracking tool, which could be easily implemented and with no additional skillsets needed as several optical modalities are routinely performed in clinical practice.

Optical imaging based on fluorophore-labeled antibodies has rapidly grown from research to clinical practice for different applications as it offers high spatial and temporal resolution, is low cost, and non-invasive^11^. Fluorophotometry, which allows the continuous measurement of fluorescence along the central axis of the eye using the blue light, was first described for assessment of the flow of the aqueous humor from cornea staining^12^ and later to investigate the in vivo permeability of the blood–vitreous barrier^13^. The technique was further improved to diagnose leakage from blood vessels associated with retinal pathology^14^ and, more recently, to measure the ocular pharmacokinetics of antibodies^15^. However, fluorophotometry lacks lateral spatial information and several tissues (lens, retinal pigment epithelium) autofluoresce at such a short wave light^16^. As David Maurice acknowledged in his exhaustive review^16^, the technique will be greatly improved if it used a fluorophore in the longer wavelength range as tissue autofluorescence will be reduced by at least an order of magnitude. Specific challenges for the clinical applications of fluorescence imaging in an ophthalmology setting need to be therefore considered. The choice of the optimal fluorescent dye for ocular applications needs to be carefully determined due to potential bleaching or quenching of the dye as well as light-scattering effects from the tissue^17^. As mentioned above, the natural autofluorescence of the retinal tissue should also be considered for the dye selection in order to avoid high background signal. The number of fluorophores per protein has to be also carefully considered because overlabeling can lead to modification of the protein distribution. Finally, a very high signal sensitivity is needed because the amount of biologics and the drug volume to be injected are limited by the microenvironment and size of the eye. An imaging-based approach using a scanning laser ophthalmoscope was previously described in rabbits^18^ and later in rats^19^ to circumvent the limitations of the fluorophotometer. We chose to perform and demonstrate a proof-of-concept approach using this technique in non-human primates (NHPs) whose eyes in terms of anatomy, optical properties, and content (collagen, hyaluronan, etc.) are closer to humans than other small species. Moreover, a readout in NHP is also needed for the development of biologics, due to potential cross-reactivity. We also standardized and expanded the technique by using an ultra-widefield lens, enabling assessment of a larger area of the tissue.

This report describes an optical imaging approach in NHP to track labeled therapeutic agents in the vitreous and the retina, en route to clinical utility. In addition to establishing the appropriate imaging parameters required for such an experiment, the differential clearance patterns of short- and long-acting agents are shown with labeled ranibizumab and a labeled long-acting anti-VEGF antibody (LAAVA).

## Results

### Initial feasibility study in cynomolgus monkeys with free IRDye800CW

**Fig. 1:**
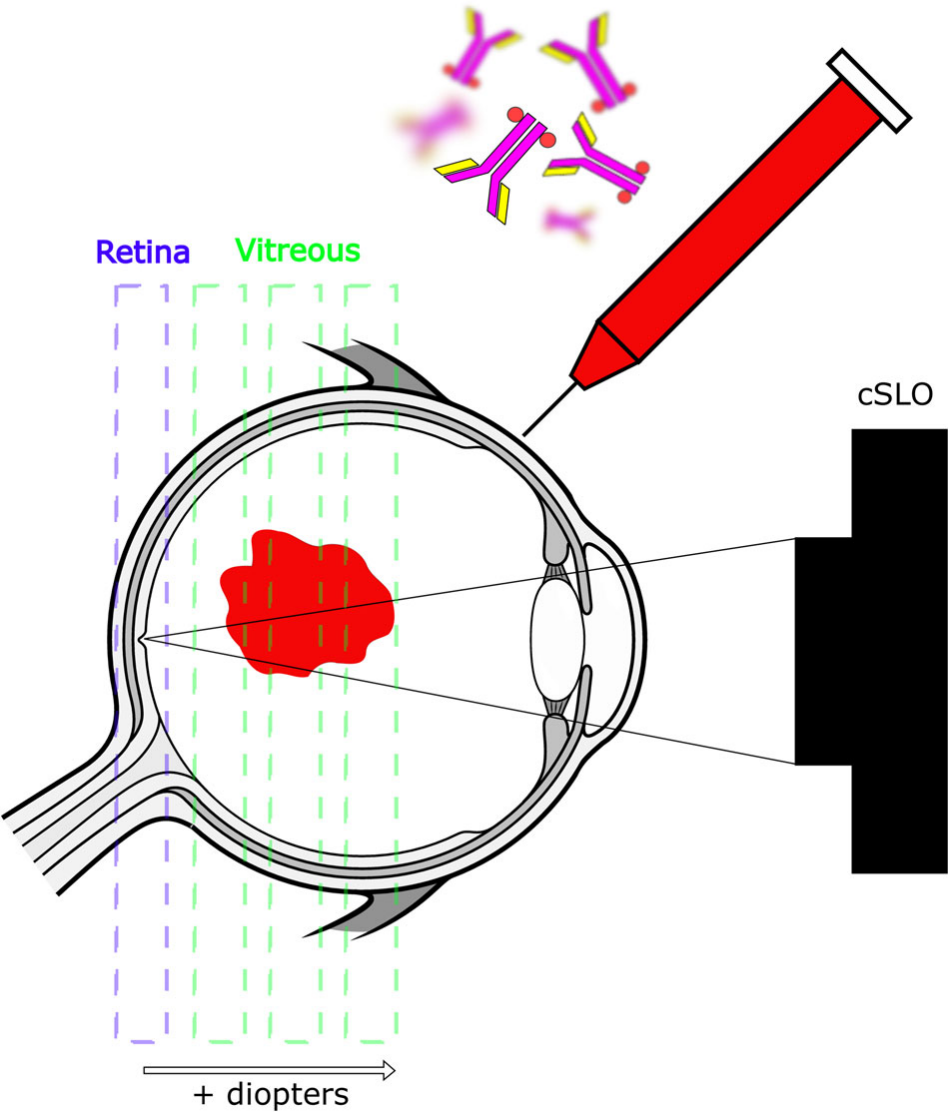
Schematic representation of the molecular tracking technology. Antibodies are labeled with an infrared fluorophore and dosed intravitreally. Immediately after injection and subsequently, a confocal scanning laser ophthalmoscope is used to quantify the distribution of the antibodies at the retina and throughout the vitreous using different focal planes. Eye design under license from https://www.Shutterstock.com.

**Fig. 2:**
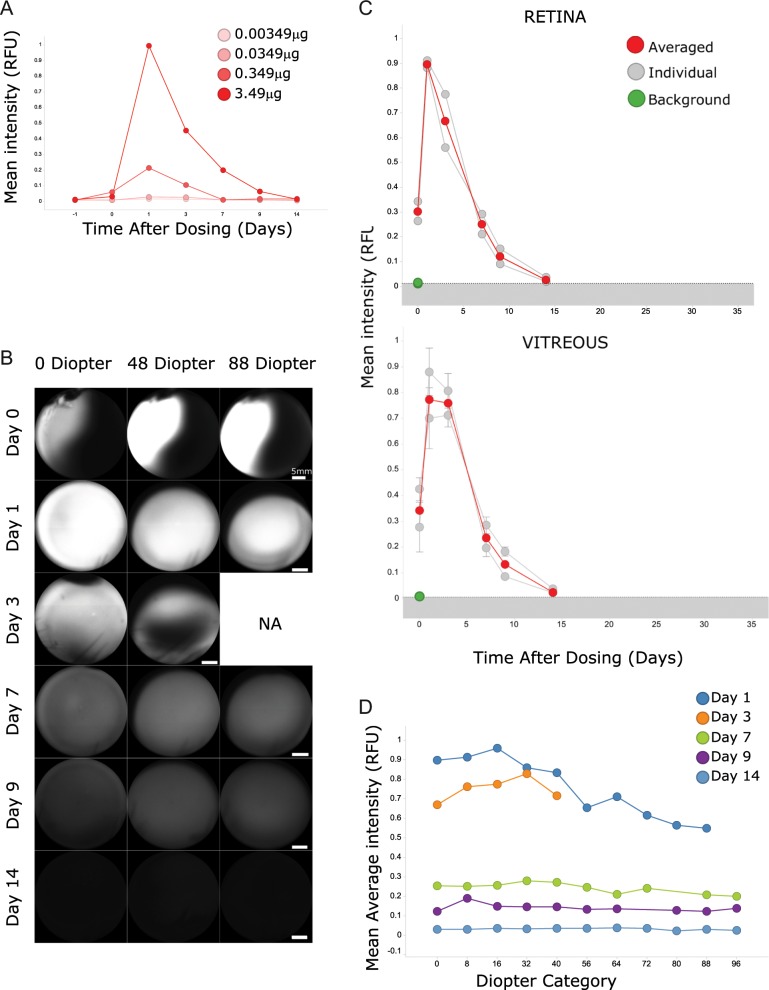
Characterization of free IRDye800CW after IVT dosing in cynomolgus monkeys. **a** Profile curves of free IRDye800CW at different doses and at different time points after IVT dosing using the 30° lens and at 60% sensitivity (each dose was tested in one eye). **b** Representative in vivo images of free IRDye800CW at different time points and diopters after IVT dosing using the 102° lens (60% sensitivity). NA image not available. Scale bars represent 5 mm. **c** Distribution profiles of free IRDye800CW per animal (n = 2) and averaged at the retina (top) and in the vitreous (bottom) using the 102° lens (60% sensitivity). For these calculations, the retina is defined in these studies as the section at 0 diopter. For the vitreous calculations, a global average across seven sections is presented (16–96 diopters). Control PBS is represented as the dotted line, which delimits the background noise area (in gray) while baseline (3 eyes at the retina and 4 eyes at 21 diopters in the vitreous) is represented as green. Error bars represent the standard deviation between the diopters in the vitreous for each individual curve. **d** Mean fluorescence of free IRDye800CW across diopters at each day using the 102° lens (60% sensitivity) for *n* = 2 animals. At Day 3, images at high diopters were not acquired.

The labeling strategy was focused on near-infrared (NIR) fluorescence to allow detection of the dye at deeper level as well as to avoid interference with the autofluorescence emitted from the retina^20^. IRDye800CW was selected as it has been shown to be less challenging for chemical reactions than indocyanine green (ICG) and quenches at higher concentration (Supplementary Fig. 1) and as seen elsewhere^21^. Furthermore, toxicology studies were conducted for this agent^21^ before use in humans for cancer research^22,23^ thus ensuring feasibility to translate to man with lesser regulatory burden. Figure 1 shows a schematic of an IVT injection and the experimental set-up to track labeled antibodies in the NHP eye. An initial set of experiments was performed to assess the feasibility of longitudinal tracking the free IRDye800CW dye in NHPs and to test the minimum detectable quantity of the injected dye in the clinical infrared system used. As seen in Fig. 2a, the free IRDye800CW dye was detected by the indirect ophthalmoscope at injected amounts of 3.49 and 0.349 µg but not <0.349 µg. Immediately after IVT injection, a highly concentrated fluorescent agglomerate was observed with clear contours and located temporally at the highest diopters, away from the retina as shown in Fig. 2b (first row). The fluorescence was also detected at the retina but at lower intensity than in the vitreous (Fig. 2b, Day 0, 0 diopter). Subsequently, the free dye seemed to homogenize at 24 h post-IVT dosing (Fig. 2b, Day 1). The fluorescence intensity of the free dye progressively declined to background level, equivalent to the phosphate-buffered saline-injected eye, at 14 days post-IVT both at the retina and in the vitreous (Fig. 2b, c). Interestingly, we observed higher fluorescence patterns at the optic nerve and at the fovea as well as punctuate fluorescent signal along the retinal blood vessels at later time points, suggesting that the labeled material may be cleared through different exit points (Supplementary Fig. 2). Figure 2d represents the variation of dye intensities across different diopters over time. Pharmacokinetic analysis showed that the free IRDye800CW exhibited a half-life of 2.68 and 2.66 days in the vitreous and retina, respectively.

### Proof-of-concept study with labeled ranibizumab

**Fig. 3:**
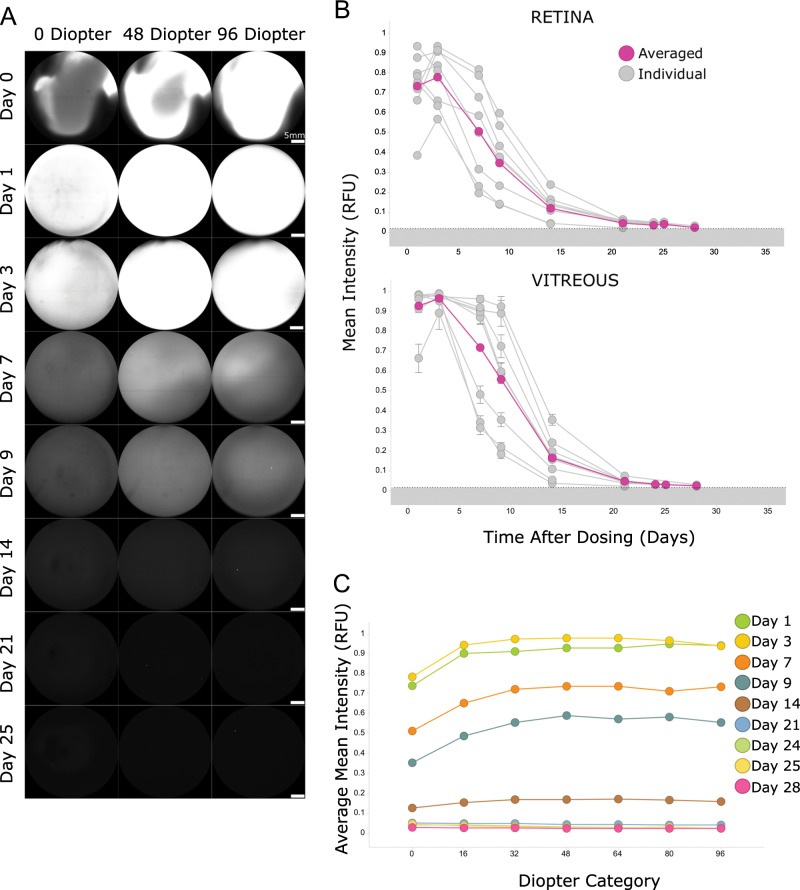
Distribution of IRDye800CW-labeled ranibizumab after IVT dosing in cynomolgus monkeys. **a** Representative in vivo images of IRDye800CW-labeled ranibizumab at different time points and diopters after IVT dosing using the 102° lens (60% sensitivity). Scale bars represent 5 mm. **b** Distribution profiles of labeled ranibizumab per animal and averaged at the retina (top) and in the vitreous (bottom) using the 102° lens (60% sensitivity). For these calculations, the retina is defined in these studies as the section at 0 diopter. For the vitreous calculations, a global average across seven sections is presented (16–96 diopters). Control phosphate-buffered saline is represented as the dotted line, which delimits the background noise area (in gray). Error bars represent the standard deviation between the diopters in the vitreous for each individual curve (*n* = 8). **c** Mean fluorescence from labeled ranibizumab at each diopter level and at different days (*n* = 8).

Based on the results above with free IRDye800CW, we labeled ranibizumab with the fluorescent dye to measure the distribution of a known Fab format in vivo. Figure 3a shows representative imaging sections from one representative NHP at different days and spatial diopters. As shown with the free dye, a fluorescent agglomerate was observed immediately after IVT injection and the fluorescence was maximal mostly at the highest diopters corresponding to the site of injection (Supplementary Fig. 3B). The IRDye800CW-labeled ranibizumab homogenized quickly after IVT injection and the fluorescence progressively declined until Day 25 after dosing for all animals (Fig. 3b). It also became apparent that not only was labeled ranibizumab detectable in the retina and vitreous but it also remained in the eye longer than the IRDye800CW alone (Figs. 2c and 3b). The decay of the fluorescence signal overtime across all diopters was evaluated to appreciate the dynamics of the assay as a whole where until Day 14 there was less fluorescence detected at the retina than in the vitreous (Fig. 3c).

### Kinetics of labeled ranibizumab in different compartments

**Fig. 4:**
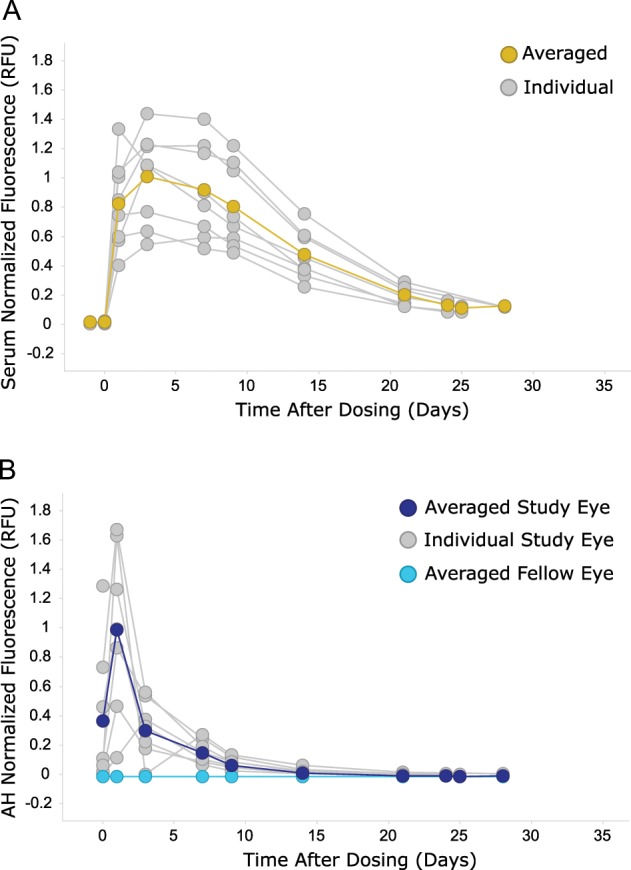
Systemic and aqueous humor exposure of IRDye800CW-labeled ranibizumab after IVT dosing in cynomolgus monkeys. Serum (**a**) and aqueous humor (**b**) samples were collected at each imaging session and analyzed by the Odyssey infrared imager to measure the fluorescence amount in each sample (*n* = 8 animals and *n* = 7 animals for serum and for aqueous humor, respectively). In order to compare ranibizumab distribution in both compartments, fluorescence was normalized to the peak of average fluorescence across samples. Background fluorescence in serum was determined at baseline (Day −1). Fluorescence from aqueous humor in the study eye (IRDye800CW-labeled ranibizumab) was compared with fluorescence from aqueous humor in the fellow eye (phosphate-buffered saline).

Because fluorescence can be easily and directly detected in any medium without any further processing and therefore loss of signal, the distribution of labeled ranibizumab was determined in other compartments ex vivo. Aqueous humor and serum samples were thus collected at the same time points as the imaging sessions after IVT dosing. As the amount of fluorescence detected cannot be directly compared between aqueous humor and serum due to the different instrumentations, results were normalized to the peak of averaged fluorescence values (Fig. 4). Interestingly, labeled ranibizumab seemed to behave differently in the two compartments: fluorescence intensity measured in serum peaked at Day 3 and slowly decreased to reach a plateau at Days 25–28 (Fig. 4a) while the fluorescence intensity measured in the aqueous humor peaked earlier (Day 1) and decreased sharply to background noise after Day 14. The kinetics of labeled ranibizumab in the serum appeared quite similar to the vitreal kinetics in terms of a peak concentration at Day 3 and a plateau at Days 25–28. However, half of the signal was detected at about Day 10 in the vitreous as compared to Day 15 in the serum, suggesting a slower clearance rate in the serum. It should be noted that we did not see any difference in terms of kinetics in animals with or without aqueous humor sampling in this small study. Hence, even though it is very possible that such sampling may affect the clearance, in this study the effect was likely not remarkable.

### In vivo distribution of a long-acting anti-VEGF antibody in the eye

**Fig. 5:**
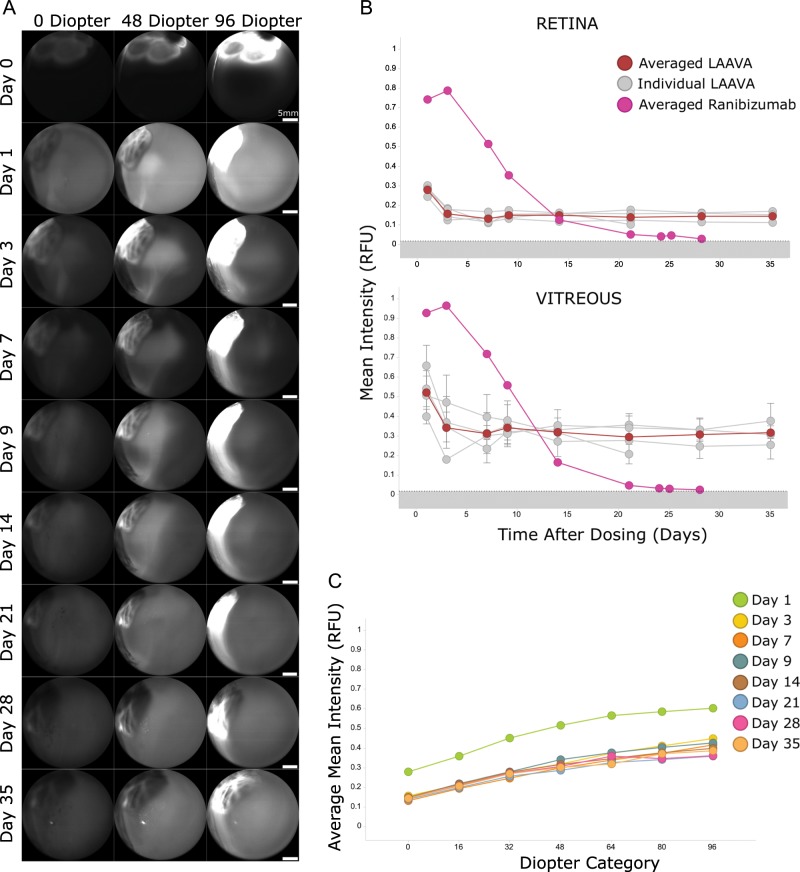
Distribution of IRDye800RS-labeled LAAVA after IVT dosing in cynomolgus monkeys. **a** Representative in vivo images of IRDye800RS-labeled LAAVA at different time points and diopters after IVT dosing using the 102° lens (60% sensitivity). Scale bars represent 5 mm. **b** Distribution profiles of labeled LAAVA per animal (*n* = 4) and averaged at the retina (top) and in the vitreous (bottom). The profiles are compared with averaged labeled ranibizumab. Control phosphate-buffered saline is represented as the dotted line, which delimits the background noise area (in gray). Error bars represent the standard deviation between the diopters in the vitreous for each individual curve. The retina is an individual diopter. **c** Mean fluorescence from labeled LAAVA at each diopter level and at different days (*n* = 4).

The results above indicate that antibodies can be tracked non-invasively and longitudinally in large animals and potentially in humans, which will enable personalized therapeutics titration in the eye. We wanted to further characterize the technique by labeling an internal LAAVA construct designed to reside longer in the vitreous than current antibodies and developed for retinal diseases^6^. Immediately after IVT injection, the labeled LAAVA showed the same agglomerate pattern as ranibizumab or IRDye800CW alone, but in contrast to the other two products, it never homogenized within the vitreous after injection (Fig. 5a). Indeed, the injection agglomerate remained, after 24 h post-IVT, at the same location throughout the study with fluorescence intensity slowly decreasing but still high at Day 35 as shown by the camera saturation. The mean fluorescence of labeled LAAVA in the vitreous at Day 1 was lower than the mean fluorescence of labeled ranibizumab, due to the persistent heterogeneity of the fluorescent signal of LAAVA, with many saturated pixels in the agglomerate and mostly low-intensity pixels in the rest of the field of view (Fig. 5b). This difference was even more pronounced for labeled LAAVA at the retina as only a small amount of it was able to reach the retina. However, the mean fluorescence of labeled LAAVA remained stable up to Day 35 and surpassed the mean fluorescence of labeled ranibizumab after Day 14. Figure 5c shows that fluorescence emitted by labeled LAAVA decreased from Day 1 to Day 3 at all locations within the vitreous and at the retina but remained stable over time with higher concentrations further away from the retina and closer to the point of injection in the vitreous.

**Fig. 6:**
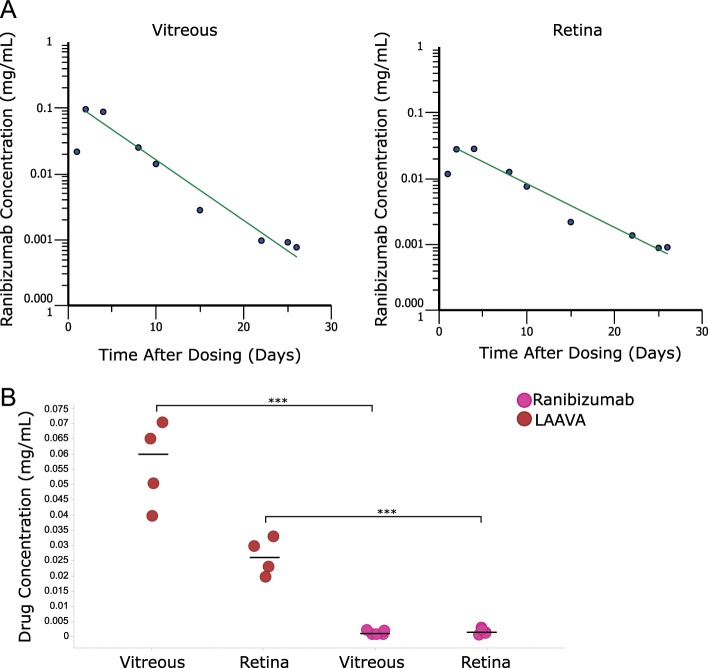
Quantitative measurements of drug concentration for pharmacokinetics and comparison of ranibizumab and LAAVA. **a** Fluorescence from labeled ranibizumab was converted into drug concentration based on concentration curves. Pharmacokinetics in both vitreous and retina were then determined by non-compartmental analysis based on mean concentrations at 40% intensity. **b** Ranibizumab and LAAVA at Day 21 post-IVT. Drug concentrations were measured in the vitreous and at the retina using concentration curves from labeled ranibizumab and labeled LAAVA. ***P < 0.0001, unpaired *t* test.

Finally, the fluorescence detected from the imaging sessions was converted into drug amount of proteins using calibration curves for each construct (Supplementary Fig. 4). Maximal ranibizumab concentrations were estimated to reach 0.1 and 0.028 mg/mL in the vitreous and retina, respectively (Fig. 6a). Further pharmacokinetic analysis showed that labeled ranibizumab exhibited a half-life and an area under the curve (AUC) of 4.52 days and 0.22 day × mg/mL in the retina. In the vitreous, the calculated half-life, AUC, clearance, and volume distribution were 3.34 days, 0.57 day × mg/mL, 0.44 mL/day, and 4.78 mL, respectively. We then compared the concentration of ranibizumab and LAAVA at Day 21 in the vitreous and at the retina. Ranibizumab concentrations were almost not detectable, whereas LAAVA concentrations were estimated at 0.057 and 0.026 mg/mL in the vitreous and at the retina, respectively (Fig. 6b).

## Discussion

The current study establishes the feasibility of an ophthalmoscope-based methodology in a large animal species, relevant to humans by optimizing experimental parameters using a clinically available device (labeling of the dye, lens needed, sensitivity setting, etc.) as well as standardizing image acquisition and analysis. The use of the ultra-widefield lens for image acquisition is especially important for long-acting agents as they may remain concentrated at the injection site in the vitreous and slowly diffuse toward the retina. We have indeed seen in some cases where the agglomerate was peripheral and not detected by the 30° lens. This imaging approach was made more robust for quantitative assessments by focusing on the retina as an anatomical landmark, which allowed comparison of images between sessions. Even though the procedure of IVT injection was standardized between animals, we observed that the injection site could vary slightly in the vitreous immediately after dosing (Supplementary Fig. 3), which may explain small differences in clearance rates between animals, given that injection sites proximal to the anterior chamber may lead to faster clearance through the anterior pathway^24^. We show here experimentally that upon IVT injection, which is delivered well into the vitreous and far away from the lens, the maximal fluorescent signal is typically obtained at the highest diopters confirming that our farthest sections often correspond to the site of the IVT injection and hence are still well within the vitreous. As a comparator, a fluorophotometric approach has similar potential^15^ but it lacks the lateral spatial discrimination needed to evaluate agents in discrete compartments of the eye. This limitation holds true for the homogenization phase of any molecule immediately after injection or highly sequestered agglomerates like the LAAVA shown here, where one line scanning would not accurately capture the overall distribution of the material in the vitreous. In addition, fluorophotometric tools often use the green channel, which overlaps with the autofluorescence of the retina, making retinal concentration measures difficult and inaccurate. Hence, a red-shifted dye was used in this study to avoid this issue. It is important to note that, in our settings (60% sensitivity and no image averaging), there is no detectable NIR signal at the wavelength used without any dye. This is consistent with results reported by Basile et al.^18^. However, attempts have been made in the past to image at the NIR frequency with high signal averaging, high sensitivity, and no eye movement^25–27^. Keilhauer et al. concluded that the NIR signal is 60–100 times weaker than the fundus autofluorescence wavelength, which suggests that the NIR is indeed the most optimal wavelength for this technique.

**Fig. 7:**
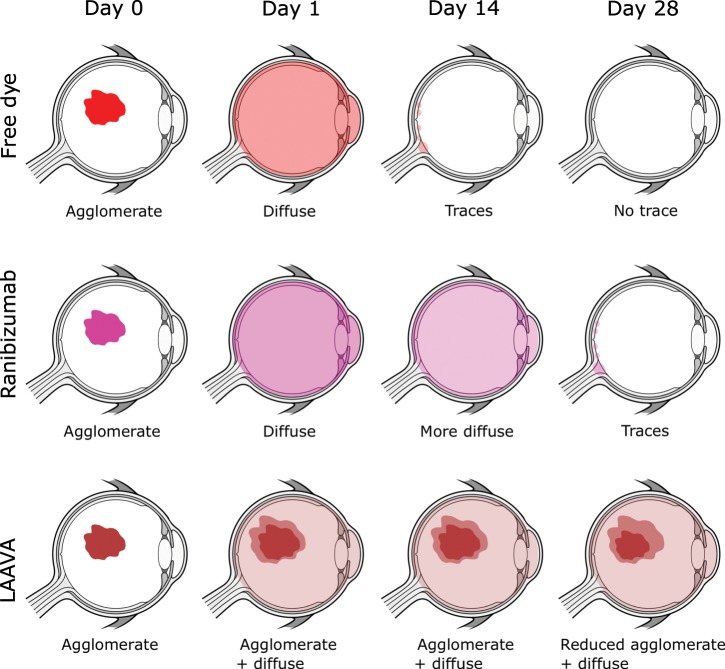
Schematic summarizing the results of this study in cynomolgus monkeys. From the non-invasive technique, we could image an agglomerate immediately after IVT injection for all agents (Day 0). Twenty-four hours post-dose (Day 1), free IRDye800CW and IRDye800CW-labeled ranibizumab were fairly homogenous while IRDye800RS-labeled LAAVA was still very concentrated in an agglomerate with faint product around it. The free dye was almost non-detectable 15 days post-IVT while ranibizumab was still present and LAAVA was mostly concentrated in the original agglomerate. At Day 28, ranibizumab was only present as traces in the vitreous and at the retina while the distribution of LAAVA remained similar from previous time points although the agglomerate was slightly shrunk. Eye design under license from https://www.Shutterstock.com.

This study also showed that tracking of labeled antibodies can yield valuable insights for drug development and may enable rapid lead optimization of new candidates, especially for complex platforms like LAAVA. For example, ranibizumab initially took few days post-IVT to equilibrate in the eyes. Over that initial window, images acquired showed variability both in fluorescence intensity and in image features. We may postulate that this first phase may be the case in man as well, although this has not been demonstrated yet. The data from Day 3 onwards were less variable and quite consistent across animals, which, when taken together with the aqueous humor data (Fig. 4), suggest that the initial diffusion may be predominantly driven by the anterior route. Beyond Day 3, as the drug equilibrates throughout the vitreous, the posterior route may be the predominant clearance route and therefore less variable between animals as labeled ranibizumab was almost cleared from the anterior chamber. Over the course of the next few days, there was a systematic decrease in the labeled molecule in the eye across both vitreous and retina (Fig. 7). It was possible to detect the presence of ranibizumab until Day 25, which is similar to other reports^8^. The pharmacokinetic analysis for ranibizumab showed a half-life of 3.34 days in the vitreous, which is consistent with half-life measured in NHPs (2.6–4 days^5,8,28^) and in humans for ranibizumab^29^ with invasive measures. By contrast, the half-life measured in the retina with this technology was higher than reported elsewhere (4.52 days as compared to 2.6–2.3 days from ref. ^5^). However, the fluorescence pattern observed in the macular area and characterized by higher concentration at the optic disc, fovea, and retina vessels at all time points suggest that drug concentration derived from tissue extraction of the entire retina may not reflect the heterogeneity in different parts of the retina. Interestingly, the maximal drug concentration and AUC calculated in the vitreous and at the retina were similar to results from ref. ^5^, when adjusted for the dose amount injected (0.25 mg). Clearance and volume of distribution were also similar between both approaches (0.594 mL/day for clearance and 2.75 mL for volume of distribution from enzyme-linked immunosorbent assay (ELISA) as compared to 0.44 mL/day and 4.78 mL from ocular tracking). The previously reported threefold vitreous:retina ratio was also observed using our technology.

Finally, we were able to compare the behavior of ranibizumab in different compartments based on imaging and sampling. Serum data revealed similar kinetics to the retina and the vitreous (that is peak concentrations at about Day 3), except for the clearance that appeared slower. Ranibizumab concentration in the aqueous humor not only peaked at Day 1 post-IVT as found by others in patients (Fig. 4a)^30^ but also sharply decreased as compared with the other compartments. This is in contrast to a previous study in NHP where the team found that half-lives of ranibizumab in aqueous humor and vitreous were identical^5^.

The imaging data from the LAAVA construct showed a very different diffusion profile in the vitreous (Fig. 7). In this case, the initial bolus remained almost intact during the entire course of the 36-day study. The drug slowly leached out of the initial bolus and the fluorescence signal outside of the agglomerate stabilized across the vitreous and retina. Moreover, the mean fluorescence of labeled LAAVA remained stable up to Day 35 and surpassed the mean fluorescence of labeled ranibizumab after Day 14 post-IVT. As the focus of IVT-dosed anti-VEGF therapies increasingly moves toward long-acting agents, this information can be used in real time for dose optimization.

The current study was done with only a single dose for initial proof of concept, but the technology can easily be adapted to study multiple doses. The dosing frequency can be thus directly adjusted based on a live measurement of the fluorescence concentrations in the eye and ensuring that a certain minimal concentration is maintained. This study was conducted in healthy and young cynomolgus monkeys, which is the closest species to mimic a healthy human eye. However, a disease status by itself (for example, neovascular age-related macular degeneration) could affect the distribution and clearance of IVT-injected biologics as could the vitreal liquefaction resulting from aging^31,32^. Interrogation of such specific pathophysiology context would require that this experiment be performed with appropriate animal models. This technology offers another unique opportunity to study pharmacodynamics and pharmacokinetics non-invasively in the same study, gaining valuable insights in the mechanism of action of a potential new drug. With optimization of labeling agents and subsequent instrumentation, one could adapt this approach for studying the distribution of topically applied drugs.

There are few limitations of the technology as described here. The quantitation of the biologics in absolute concentration remains a challenge as the concentration calibration curves for reference can only be generated outside the eye. Despite this caveat, the estimated concentrations in the different tissues calculated using this technology were similar to reported concentrations by other colleagues using ELISA assays^5^. Moreover, images with saturating pixels within an agglomerate will lead to an underestimation of the true amount of the fluorescence. This can be avoided by either reducing the sensitivity of the camera or reducing the number of fluorophores per protein. The second limitation is the fact that, even though the ultra-widefield lens covered a higher surface of retina and vitreous, the measured fluorescence may not capture the entire retina or vitreous, limiting the calculations of concentration in the total volume of the eye. Furthermore, the instrument used in this study is not a true pinhole confocal microscope, therefore anatomical sectioning is not accurate. Similarly, because the presence of the drug in the different layers cannot be discerned, the imaging at the retina is an integrated measurement of the drug distribution rather than specific to a retinal layer. One possible strategy to circumvent this limitation will be to use the optical coherence tomographic range and software that could detect small amounts of optical coherence tomographic contrast agents in the different layers based on a reference baseline. Finally, the labeling strategy described does not allow the differentiation of bound and free antibody, which is critical as binding activity will modulate the residency time.

This technique was first described in rabbits to compare the kinetics of the free dye with research antibodies^18^, which therefore lacked a reference antibody to compare this technology with invasive techniques. More recently, ocular tracking was tested for VEGF imaging using labeled bevacizumab in a rat laser-induced neovascularization model^19^. The team reported several hyperfluorescent spots, which prevented interpretation of results and were thought to be related to an immune response in this species. The absence of hyperfluorescent spots in NHP seems to corroborate this hypothesis. However, we also observed hyperfluorescent speckles lining the major vessels for both free dye and labeled antibodies, suggesting a common mechanism for clearance in both species. The speckles in the posterior blood vessels, while indicative of the presence of the compound, may allude to a clearance path, but it is not possible within the scope of this work to estimate how much of the total clearance is governed by this path. The aim of this current study was to standardize and further develop the methodology in NHP to enable direct data generation of biotherapeutics in a clinically more relevant species without additional regulatory burden or devices. With additional needs, this set-up could be translated to man, although the fluorophore-bound material may need prior health authority approval. However, the IRDye800CW has been used in man for research studies^22,23^, and this may offer faster translational path to the clinic.

In conclusion, we developed a novel imaging methodology in cynomolgus monkeys, which allows longitudinal molecular tracking of IVT-injected molecules. This methodology could allow direct monitoring of any IVT-injected therapies and help decide whether the agent needs to be re-injected to sustain efficacy^33^.

## Methods

### Reagents

IRDye800CW (LI-COR Biosciences; Lincoln, NE) was chosen as the NIR dye with optimal characteristics needed for translation and signal intensity. It was reconstituted in phosphate-buffered saline as 34.9 µg/mL to inject 3.49 µg for IVT. Labeling of ranibizumab and LAAVA antibodies with IRDye800CW and IRDye800RS, respectively, was performed by LI-COR using *N*-hydroxysuccinimide conjugation. Briefly, the antibody was removed of preservatives by passing it through a desalting column and prepared at a concentration of 5–10 mg/mL. The *N*-hydroxysuccinimide ester dye was then added to the antibody at a ratio of 4:1 and 2:1, respectively, and incubated for 2 h at 20 °C. The free dye was separated from the labeled antibody by using desalting Zeba columns (ThermoFisher; Waltham MA) and samples were aliquoted as 5 mg/mL in 1× phosphate-buffered saline. Free dye amount was determined to be 0.7% in the labeled ranibizumab by high-performance liquid chromatography and <0.5% in the labeled LAAVA by quantitative sodium dodecyl sulfate-polyacrylamide gel electrophoresis. The dye-to-protein ratio was calculated as 2.0 and 1.4 for ranibizumab and LAAVA, respectively, by ultraviolet–visible spectroscopic analysis. The imaging agents were injected IVT (volume: 50 µL per eye; final injected protein dose for both labeled ranibizumab and labeled LAAVA was 0.25 mg).

### Animals

Studies were conducted in male cynomolgus monkeys (*n* = 13, age 5–8 years) according to a protocol approved by the Novartis Animal Care and Use Committee. On the days of imaging, the animals were anesthetized by intramuscular injection of ketamine, and xylazine or midazolam and pupillary dilation was induced topically with two drops each of tropicamide (0.5%, Akorn, Lake Forest, IL) and phenylephrine (2.5%, Paragon Bio-Tech Inc., Portland, OR) in each eye. If needed, application of additional drops was given to allow further pupil dilation. A lubricant (Genteal; Alcon, Fort Worth, TX) was topically administered throughout the session to maintain hydration.

### IVT injection

During the procedure, ordinary aseptic rules were respected to prevent bacterial contamination of tissues. A sterile 1-cc syringe and 30-gauge needle were used for each injection. Phosphate-buffered saline solution was used as control in the contralateral eye. IVT injection was performed at the pars plana between 3 and 4 mm from the limbus cornea. The needle was inserted to the hilt through the conjunctiva, if possible off center, and through the sclera, perpendicular to the eye wall, aiming toward the center of the globe. The agent was injected slowly until all the material was delivered, and after few seconds, the needle was removed slowly to prevent any reflux. A topical antibiotic (Tobramycin, Henry Schein, Melville, NY) was applied in each eye after injection.

### Imaging

A standard clinical confocal laser ophthalmoscope (cSLO/Spectralis; Heidelberg, Germany) was used for all studies. An ultra-widefield lens (~102°) was used to get a pan view of the entire retina while a 30° lens was used to focus imaging at the macula. Our experiments used 780 nm NIR fluorescence excitation light with the NIR observation setting set at 800 nm. Images were acquired using a camera sensitivity from 40% to 90% (unless mentioned otherwise, all imaging reported here was performed at 60%). The camera was focused on the retina using the infrared reflectance mode for anatomical alignment. Once aligned, the imager was switched to the NIR mode (ICG), and images were acquired along different sections of the vitreous starting at the retina as the 0 diopter. The retina was used as the anatomical marker longitudinally to ensure inter-day alignment. Each imaging session lasted about 20 min.

### Image analysis

Images were exported as tiff files through XML export from the Heidelberg Spectralis and serially read into Matlab (Mathworks; Natick, MA) using a customized script. All images were displayed in gray scale (8 bit). A global average (mean) intensity of signal in the field of view was automatically calculated at each slice from each animal at each time point to generate a time course as well as a spatial distribution of the fluorescent signal in the eye. Both time course and spatial distribution from the retina (0 diopter) to the front of the eye (96 diopters) were plotted using Spotfire (Tibco; Palo Alto, CA). At each time point, all vitreous sections were digitally summed to represent signal from the entire imaged vitreal chamber and then it was averaged across all NHPs to yield one composite volumetric data per time point. For the retina, only the section corresponding to 0 diopter was averaged across animals at each time point.

### Blood collection and preparation

Peripheral venous blood was collected through the saphenous vein for detection of the labeled antibody or free dye in the vasculature at different time points after injection up to 35 days. One milliliter of blood was collected with Covac serum separator tubes (Medtronic; Mansfield, MA) for further preparation of ~340 µL serum. Immediately after blood collection, the tube was gently inverted a minimum of five times to ensure content mixing. The tube was then placed upright in a rack and allowed to clot for 30 min at room temperature. Following clotting, the tubes were centrifuged immediately for 5 min at 2000 × *g* force. Immediately after centrifugation, the supernatant from each sample was transferred into a pre-chilled polypropylene vial (Eppendorf; Hauppage, NY). The vial was inverted a minimum of five times to ensure mixing of tube content and then serum was aliquoted into three pre-chilled polypropylene cryovials (Corning; Corning, NY). The 1-ml cryovials containing the serum aliquots were immediately placed on dry ice. Once completely frozen, the tubes were transferred to a freezer for storage below −70 °C until used for analysis.

### Aqueous humor collection

For the aqueous humor collection at the same imaging time points, the same sterile conditions and anesthesia procedures as for an IVT procedure were used. About 50 µL of aqueous humor was collected via a limbal paracentesis and samples were immediately placed on dry ice and later stored at −70 °C until used for analysis.

### Fluorescence scanning

The fluorescence of the serum and aqueous humor samples collected were measured using an Odyssey CLX Infrared Imaging System (LI-COR Biosciences; Lincoln, NE). After thawing the samples on wet ice, 100 µL of serum and 30 µL of aqueous humor samples were plated in 96- and 384-well plates, respectively (Corning; Corning, NY). The plates were immediately imaged using different intensities.

### Data analyses

Based on concentration curves generated for each construct using Spectralis, fluorescence intensity was converted to protein concentration. A 40% sensitivity acquired during image acquisition was used here to avoid saturation of the camera and therefore reflect exact values. The half-lives of labeled antibodies in the vitreous and at the retina were then measured using a non-compartmental analysis on mean concentrations for all data points tested in each compartment using at least five time points with time range between Day 2 and last Day tested as the curve fitting approach and an uniform weighting (WinNonlin, Pharsight Corp., Mountain View, CA). From the vitreous results, clearance (*C*_L_) was calculated from the formula: *C*_L_ = dose/AUC, where dose is the amount of protein injected IVT (0.25 mg) and AUC is the area under the curve from the time of dosing to the last measurable concentration. The volume of distribution (*V*) was calculated from the equation: *V* = *C*_L_/*k*_el_, where *k*_el_ is the elimination rate constant obtained from the slope of the terminal data from the concentration–time plot. Owing to the slow clearance of LAAVA, the kinetic curve did not decrease during the study and its half-life was therefore not reported.

### Statistics and reproducibility

When shown, error bars represent standard deviation. An unpaired *t* test was performed to demonstrate significant differences between the concentration of ranibizumab and LAAVA in the vitreous and retina using Prism (version 8.1.2, GraphPad, San Diego, CA).

### Reporting summary

Further information on research design is available in the Nature Research Reporting Summary linked to this article.

## Supplementary information

Supplementary Information

Reporting Summary

## Data Availability

The authors declare that the data supporting the findings of this study are available within the paper. The datasets generated and analyzed during the current study are not publicly available due to confidential information from the two antibodies used but are available from the corresponding author on reasonable request.
