# Supplementary material for: Non-invasive molecular tracking method that measures ocular drug distribution in non-human primates
Source: Commun Biol. 2020 Jan 8;3:16. doi: 10.1038/s42003-019-0731-9 (PMC6949284; doi:10.1038/s42003-019-0731-9)
Supplement: Supplementary file 1 — Supplementary Information [file 42003_2019_731_MOESM1_ESM.pdf]

Supplementary Figures

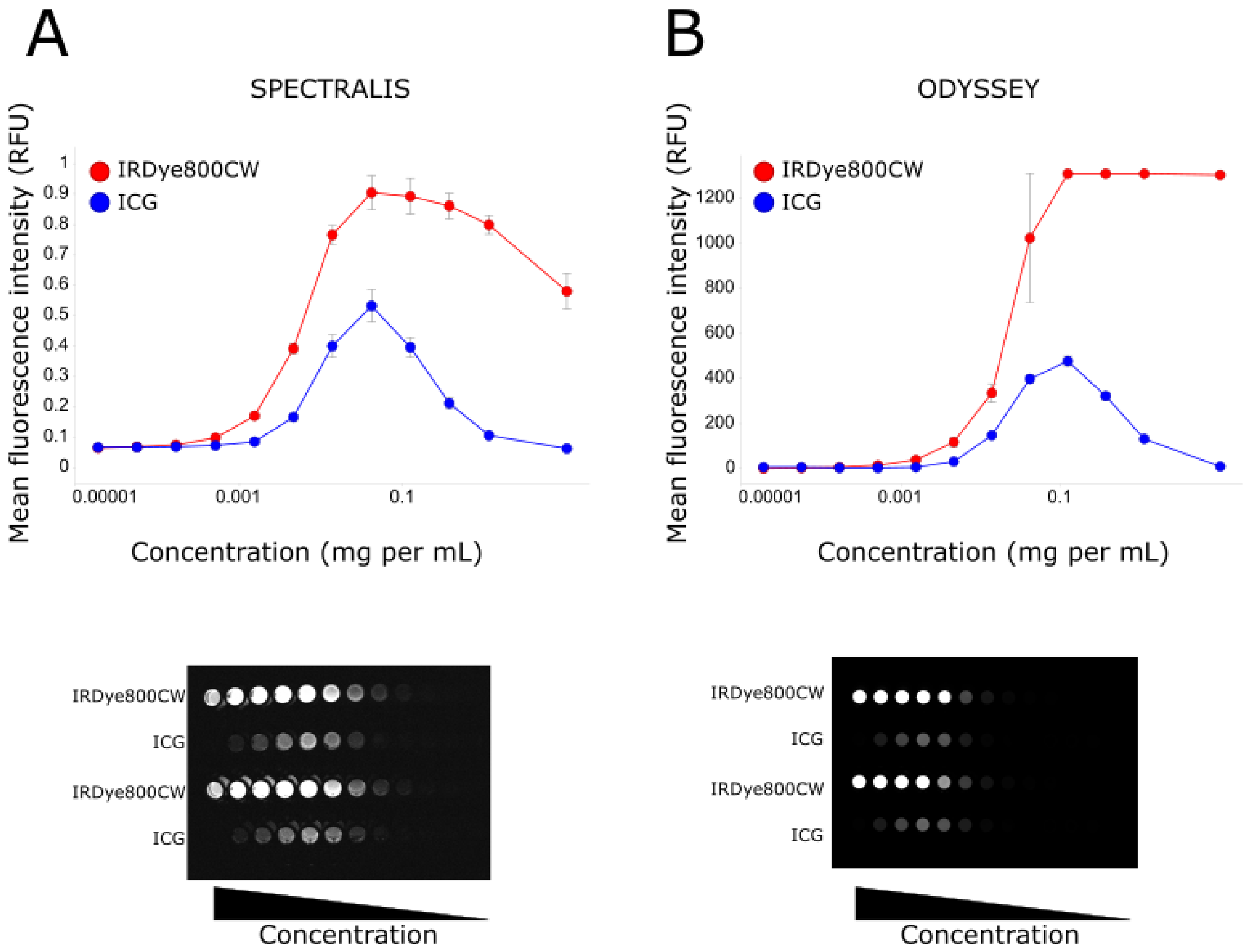

Supplementary Figure 1. **Comparison of IRDye800CW and ICG *in vitro*. A,B**. Concentration curves of ICG and IRDye800CW as measured with **A.** an indirect ophthalmoscope (Spectralis) at 80% sensitivity and **B.** a fluorescence scanner (Odyssey) at L2 intensity gain. Images below the graphs are representative images of each output. Error bars represent standard deviation of duplicate wells from two different plates.

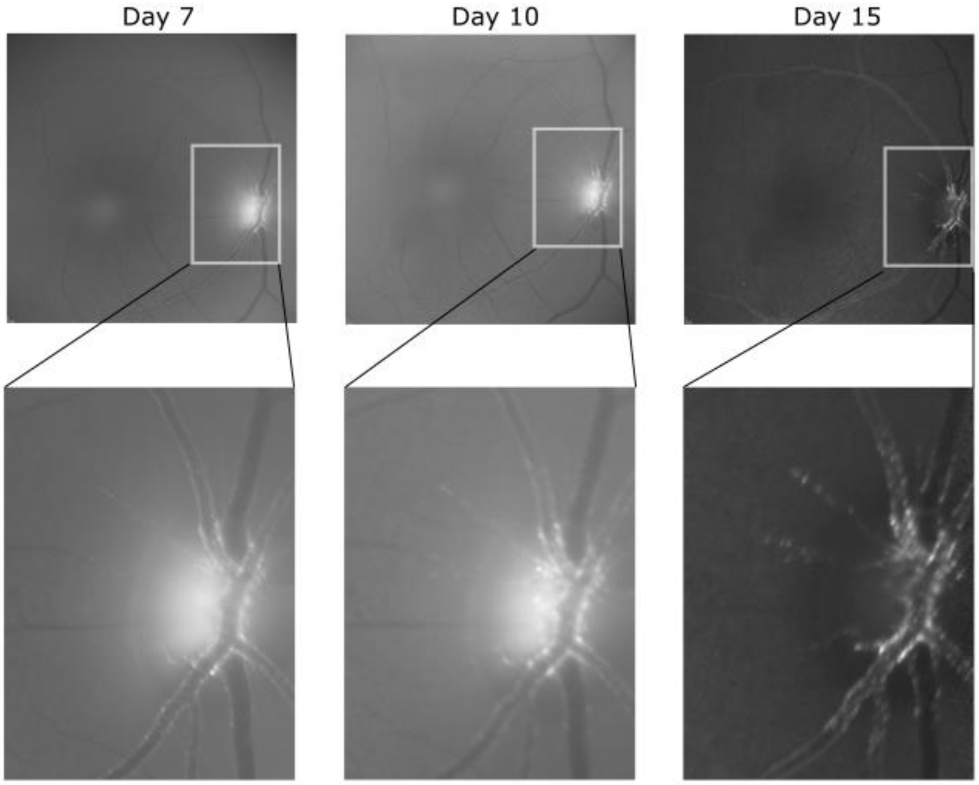

Supplementary Figure 2. Localized accumulation of IVT-injected free IRDye800CW. Examples of averaged images (ART set at 100) from free dye at Day 7, 10 and 14 using the 30° lens. Sensitivity was set differently to allow optimal signal to noise ratio. The images from the bottom row represent the magnified view of the white inserts from the top row.

A

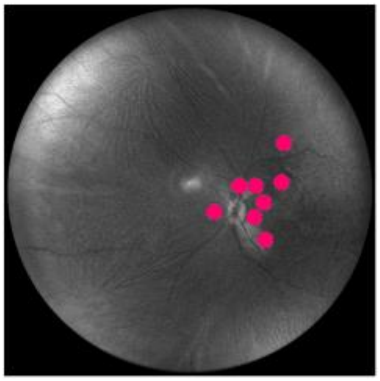

B

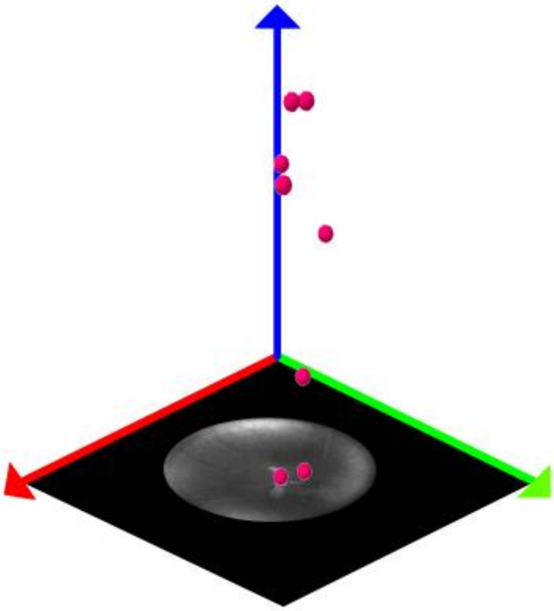

Supplementary Figure 3. Evaluation of injection sites for IRDye800CW-labeled ranibizumab. Red dots represent the locations of injection site based on the centroid of maximal pixel intensity area and were registered on a representative en-face IR image (**A**) and in 3D space reflecting the imaged vitreous (**B**).

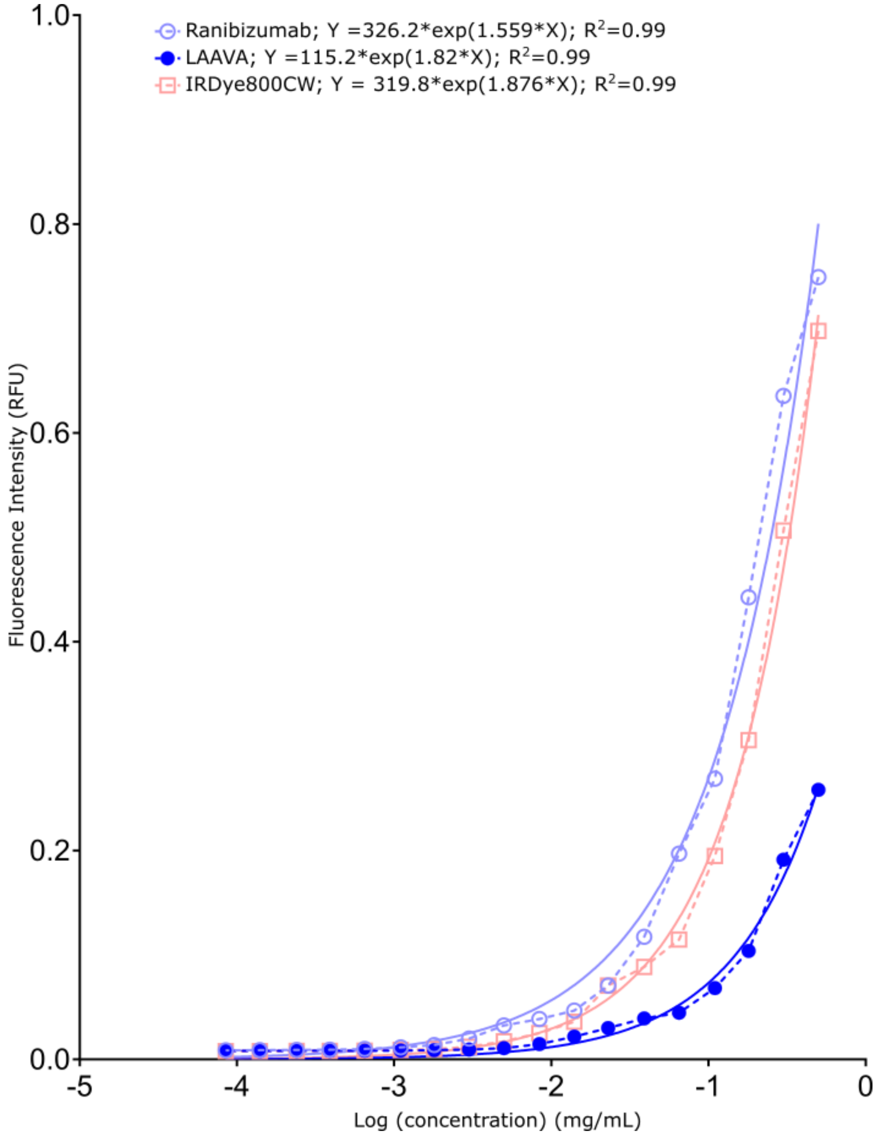

Supplementary Figure 4. Calibration curves for free IRDye800CW, IRDye800CW-labeled ranibizumab and IRDye800RS-labeled LAAVA. Different concentrations of the agents were imaged in the same conditions as *in vivo* imaging at 40% sensitivity in order to avoid saturation. Concentrations were log transformed and a non-linear fit was applied as best fit to generate the equation used to convert the fluorescence measured into concentration.
