# Supplementary material for: Non-invasive molecular tracking method that measures ocular drug distribution in non-human primates
Source: Commun Biol. 2020 Jan 8;3:16. doi: 10.1038/s42003-019-0731-9 (PMC6949284; doi:10.1038/s42003-019-0731-9)
Supplement: Supplementary file 2 — Reporting Summary [file 42003_2019_731_MOESM2_ESM.pdf]

nature research

Corresponding author(s): Guillaume Normand

Last updated by author(s): Nov 25, 2019

Reporting Summary

Nature Research wishes to improve the reproducibility of the work that we publish. This form provides structure for consistency and transparency in reporting. For further information on Nature Research policies, see Authors & Referees and the Editorial Policy Checklist.

Statistics

For all statistical analyses, confirm that the following items are present in the figure legend, table legend, main text, or Methods section.

n/a	Confirmed	
☐	☒	The exact sample size (*n*) for each experimental group/condition, given as a discrete number and unit of measurement
☐	☒	A statement on whether measurements were taken from distinct samples or whether the same sample was measured repeatedly
☐	☒	The statistical test(s) used AND whether they are one- or two-sided<br>*Only common tests should be described solely by name; describe more complex techniques in the Methods section.*
☒	☐	A description of all covariates tested
☒	☐	A description of any assumptions or corrections, such as tests of normality and adjustment for multiple comparisons
☐	☒	A full description of the statistical parameters including central tendency (e.g. means) or other basic estimates (e.g. regression coefficient) AND variation (e.g. standard deviation) or associated estimates of uncertainty (e.g. confidence intervals)
☒	☐	For null hypothesis testing, the test statistic (e.g. *F*, *t*, *r*) with confidence intervals, effect sizes, degrees of freedom and *P* value noted<br>*Give P values as exact values whenever suitable.*
☒	☐	For Bayesian analysis, information on the choice of priors and Markov chain Monte Carlo settings
☒	☐	For hierarchical and complex designs, identification of the appropriate level for tests and full reporting of outcomes
☒	☐	Estimates of effect sizes (e.g. Cohen's *d*, Pearson's *r*), indicating how they were calculated

Our web collection on statistics for biologists contains articles on many of the points above.

Software and code

Policy information about availability of computer code

Data collection	*Provide a description of all commercial, open source and custom code used to collect the data in this study, specifying the version used OR state that no software was used.*
Data analysis	A Matlab script was used to batch analyze the fluorescence in each image and consolidate analysis from different timepoints and studies to avoid manual input that could lead to errors.

For manuscripts utilizing custom algorithms or software that are central to the research but not yet described in published literature, software must be made available to editors/reviewers. We strongly encourage code deposition in a community repository (e.g. GitHub). See the Nature Research guidelines for submitting code & software for further information.

Data

Policy information about availability of data

All manuscripts must include a data availability statement. This statement should provide the following information, where applicable:

- Accession codes, unique identifiers, or web links for publicly available datasets
- A list of figures that have associated raw data
- A description of any restrictions on data availability

The datasets generated and analyzed during the current study are not publicly available due to confidential information from the two antibodies used but are available from the corresponding author on reasonable request

Field-specific reporting

Please select the one below that is the best fit for your research. If you are not sure, read the appropriate sections before making your selection.

☒ Life sciences ☐ Behavioural & social sciences ☐ Ecological, evolutionary & environmental sciences

For a reference copy of the document with all sections, see nature.com/documents/nr-reporting-summary-flat.pdf

Life sciences study design

All studies must disclose on these points even when the disclosure is negative.

Sample size	The sample size was limited due to limited access to non-human primates. The number of animals needed for each group was determined on previous experiments with the free dye and the 30 degree lens where variability of kinetics between eyes was deemed low.
Data exclusions	No data was excluded
Replication	The results for the two antibodies are from different studies and with different animals. The behavior of the two antibodies was reproduced.
Randomization	Not applicable in non-human primates
Blinding	Blinding was not possible as we could only image few animals at a time and had to disclose to our IACUC the material used.

Reporting for specific materials, systems and methods

We require information from authors about some types of materials, experimental systems and methods used in many studies. Here, indicate whether each material, system or method listed is relevant to your study. If you are not sure if a list item applies to your research, read the appropriate section before selecting a response.

Materials & experimental systems

n/a	Involved in the study	
☐	☒	Antibodies
☒	☐	Eukaryotic cell lines
☒	☐	Palaeontology
☐	☒	Animals and other organisms
☒	☐	Human research participants
☒	☐	Clinical data

Methods

n/a	Involved in the study	
☒	☐	ChIP-seq
☒	☐	Flow cytometry
☒	☐	MRI-based neuroimaging

Antibodies

Antibodies used	ranibizumab (clinical, Genentech), long-acting anti-VEGF antibody (internal antibody)
Validation	Ranibizumab is a well known anti-VEGF antibody and has been used in non-human primates for PK studies. The long-acting anti-VEGF antibody was previously used internally in rabbit for PK studies.

Animals and other organisms

Policy information about studies involving animals; ARRIVE guidelines recommended for reporting animal research

Laboratory animals	cynomolgus monkeys, male, age varies
Wild animals	the study did not involve wild animals
Field-collected samples	the study did not involve field-collected samples
Ethics oversight	The Novartis IACUC approved the protocol and USDA audits every year all animal studies performed at Novartis

Note that full information on the approval of the study protocol must also be provided in the manuscript.
